# Case report: Transcatheter aortic valve replacement in a large bicuspid anatomy using the XL-Myval 32 mm

**DOI:** 10.3389/fcvm.2022.1045280

**Published:** 2022-11-23

**Authors:** Ahmed Elkoumy, Christian J. Terkelsen, Mahmoud Abdelshafy, Julia Ellert-Gregersen, Hesham Elzomor, Troels Thim, Patrick W. Serruys, Osama Soliman, Henrik Nissen

**Affiliations:** ^1^Health Service Executive and CORRIB Research Center for Advanced Imaging and Core Laboratory, Discipline of Cardiology, Saolta Group, Galway University Hospital, University of Galway, Galway, Ireland; ^2^Islamic Center of Cardiology, Al-Azhar University, Cairo, Egypt; ^3^Department of Cardiology, Aarhus University Hospital, Aarhus, Denmark; ^4^Department of Cardiology, Odense University Hospital, Odense, Denmark; ^5^CÚRAM, SFI Research Centre for Medical Devices, Galway, Ireland

**Keywords:** bicuspid aortic valve, large annulus, transcatheter aortic valve replacement, Myval-XL, BAV

## Abstract

Transcatheter aortic valve replacement (TAVR) is a recommended intervention for selected population with severe aortic stenosis (AS). Bicuspid aortic valve (BAV) anatomy has been categorized as an unfavorable anatomy for TAVR due to multiple considerations as exclusion from randomized trials in addition to the challenging and unpredictable anatomy. The anatomical constraints of BAV include the large anatomy of the annulus, sinus of Valsalva, and aorta (aortopathy), in addition to significant calcifications of the device landing zone. Most commercial transcatheter heart valves (THV) have upper dimension limits of the annulus and area in which the device can be implanted safely without significant oversizing. Myval-XL THVs (Meril Life Sciences Pvt. Ltd., India) are balloon-expandable valves (BEV) that have been developed with two new sizes, 30.5 and 32 mm, aiming to treat patients with large annulus dimensions and that exceed the upper limit of an ordinary device’s sizing matrix. This case series report describes TAVR using the XL-Myval 32 mm THV in three European patients with symptomatic severe bicuspid aortic stenosis with significant calcifications and large annular dimensions exceeding the limits of the other THVs.

## Introduction

Transcatheter aortic valve replacement (TAVR) is a recommended intervention mode for patients with severe aortic stenosis (AS) with a tricuspid AV anatomy (1, 2). TAVR for AS and bicuspid aortic valve (BAV) disease remains challenging due to multiple anatomical and technical obstacles. BAV is considered an unfavorable anatomy for TAVR due to its exclusion from randomized trials. In addition, there is no consensus about the effectiveness of TAVR in BAV in comparison to surgical aortic valve replacement. The available evidence of TAVR in BAV has been derived from observational registries and experts’ opinions (3). A critical challenge within BAV is the relatively large aortic root anatomy, including annulus diameters, that might exceed the sizing matrix of the available commercial TAVR devices (4), in addition to the associated high calcium burden, fused raphe, and aortic dilatation. Here we present a case series of three patients with low surgical risk and severe calcific BAV stenosis with large annular dimensions, treated with the novel balloon-expandable (BE) transcatheter Myval-XL 32 mm valve. All procedural characteristics, in addition to 30-day clinical and hemodynamic outcomes, are reported to describe the performance of the large new device in such a challenging anatomy.

## Patient 1

**FIGURE 1:**
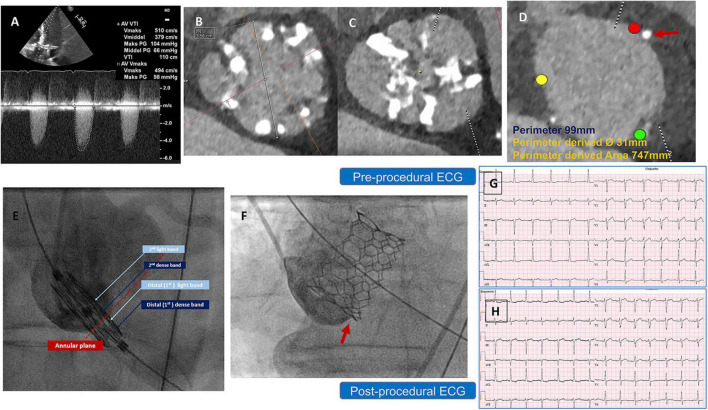
**(A)** Preprocedural AV CWD recording from Apical 5 chamber view, with high maximum velocity = 5.1 m/s, mean PG = 66 mmHg **(B)** inter commissural distance (4–5 mm) above the annular level = 35 mm, **(C)** AV significant calcification at the raphe level **(D)** annulus measurement showing small annular calcific nodule, and **(E)** illustration of the Myval design showing the dense (dark blue arrows), light bands (light blue arrows) and deployment within the annular plane (red dotted line) to obtain a proper position and implantation depth. **(F)** Implantation depth **(G,H)** pre and postprocedural ECG without changes. AV, Aortic valve, BAV, Bicuspid Aortic Valve, CCTA, Cardiac Computed Tomography, CWD, continuous wave Doppler, ECG, Electrocardiogram, PG, Pressure Gradient.

The first patient was a 76-year-old man with severe symptomatic AS. A transthoracic echocardiogram (TTE) showed a transvalvular velocity of 5.1 m/s, with maximum and mean pressure gradients (PG) of 100 and 60 mmHg, respectively. The AV effective orifice area (EOA) was 0.6 cm^2^ with mild aortic regurgitation and a reduced left ventricular (LV) ejection fraction (EF) of 35%. A preprocedural-cardiac computed tomography angiography (CCTA) confirmed a severely calcified BAV, Sievers’ type 1-a, with a calcified raphe and annular calcific nodule (5 × 5 mm). The measurements showed a very large annulus with a perimeter of 99 mm, a perimeter-derived diameter of 31 mm and an area of 747 mm^2^, wide sinuses (36 × 40 × 44 mm), inter commissural distance (ICD) 4–5 mm above the annular level of 35 mm, a sino-tubular junction diameter of 34 mm, and a mildly dilated ascending aorta of 43 mm. The left and right coronary heights were 15 and 25 mm, respectively (Figure 1). After a heart-team discussion, transfemoral (TF) TAVR using the BE Myval-XL 32 mm, was decided upon. The baseline ECG showed a normal sinus rhythm, a PR interval of 172 ms, and a QRS duration of 110 ms. The TVH was introduced through the right common femoral artery (CFA) after local anesthesia using a 14 Fr Python sheath (Meril Life Sciences Pvt. Ltd., India). Balloon pre-dilatation was performed using a Sapien-25 mm balloon (Edwards Lifesciences, LLC, CA, USA). The device was crimped over the Navigator delivery system (Meril Life Sciences Pvt. Ltd., India). The deployment within the annulus was performed in the tri-coplanar view (LAO15/CRA3), guided by the dense and light marking bands of the crimped device, a feature characteristic of the Myval (Figure 1E and Supplementary Video 1), with temporary pacing over the LV guidewire.

Post-implantation aortography and invasive transvalvular gradient were acceptable with Sellers’ grade 1 and 5 mmHg. No intraprocedural complications were reported, and the post-procedural ECG showed no new conduction disturbance (Figures 1G,H). After 2 days, the patient was discharged from the hospital on Aspirin as an antithrombotic treatment. Thirty-day follow-up documented a good clinical status, with a mean trans-prosthetic PG of 7 mmHg, an AV-EOA of 2.0 cm^2^ with trace paravalvular leakage (PVL), and a significantly improved EF to 50% (Table 1).

**TABLE 1:** Thirty-day follow-up for each of the three patients.

	Patient 1	Patient 2	Patient 3
Pacemaker implantation	No	No	No
Renal impairment	No	No	No
Body surface area, m^2^*	2.18	2.31	2.39
Body mass index, kg/m^2^	35.1	32.8	30.4
**TTE-follow up**			
Trans prosthetic Mean PG, mmHg	7	4	7
EOA, cm^2^	2.0	^†^	2.7
EOA index	0.92^‡^		1.1
PVL	Trace	Trace	Trace
LV-EF%	50	50	55

*The three patients have relatively large BSA and high BMI. ^†^EOA was not measured due to the missing mandatory parameters for the continuity equation. ^‡^EOA index is acceptable regarding the patients’ high BMI. TTE, Trans thoracic echocardiogram; PG, Pressure gradient; EOA, Effective orifice area; PVL, paravalvular leakage; LV-EF, Left ventricle-ejection fraction.

## Patient 2

**FIGURE 2:**
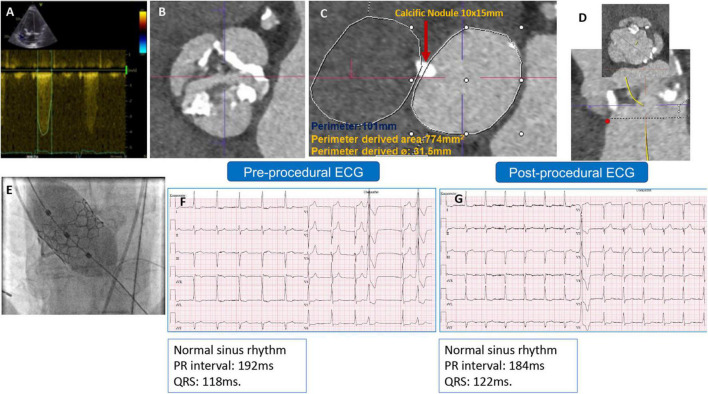
**(A)** Preprocedural AV continuous wave Doppler with high maximum velocity, **(B)** preprocedural MSCT showing BAV with partially fused L-R raphe and significant AV calcifications, **(C)** annulus measurement showing large annulus with large annular calcific nodule, **(D)** LVOT profile view (no calcification) and ICD 4–5 mm above the annulus = 35 mm, **(E)** Myval-XL 32 mm deployment, **(F)** preprocedural ECG, and **(G)** postprocedural ECG without changes.

The second patient was a 79-year-old man with severe AS. A TTE revealed a maximum and mean PG of 71 and 54 mmHg, respectively, an EOA of 0.7 cm^2^, and a preserved LV-EF. A preprocedural-CCTA showed a calcified BAV Sievers type 1-a, with a large annular calcific nodule (10 × 15 mm). The measurements documented a large annulus with a perimeter of 101 mm, a perimeter-derived diameter of 31.5 mm, an area of 774 mm^2^, an ICD of 35 mm, and wide sinuses (Figure 2). A 32 mm Myval-XL was selected for TF-TAVR with the risk of annular injury due to the annular calcium chunk (not extending to the LVOT). Balloon pre-dilatation was performed using a Sapien-25 mm balloon. The device deployment was performed using the Navigator delivery system in the tri-coplanar view (RAO4/CRA5) with pacing over the LV guidewire. Final aortography (Supplementary Video 2) and invasive transvalvular gradient revealed Sellers’ grade 0 and 5 mmHg. The post-procedural ECG showed no alteration of the conduction (Figures 2F,G). The patient had an uneventful in-hospital course and was discharged on day 2 with antiplatelet monotherapy (Aspirin). The patient’s 30-day outcomes were uneventful (Table 1).

## Patient 3

**FIGURE 3:**
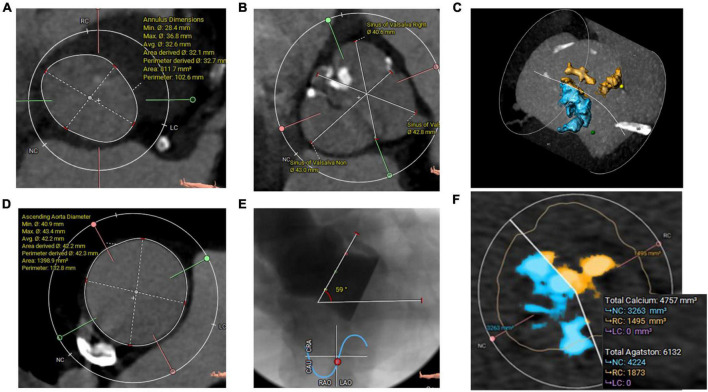
**(A)** CCTA Annulus measurements with a very large area, **(B)** wide sinuses, **(C)** all calcifications (3D Hockey Puck-MIP view), **(D)** ascending aorta diameters, **(E)** angiographic aortic angulation showed horizontal aorta, and **(F)** aortic valve calcifications (Agatston and volume mm^3^) measured from non-contrast CT scan.

The third patient was a 69-year-old man with symptomatic severe AS and a history of paroxysmal atrial fibrillation and coronary intervention. A TTE confirmed severe AS with an EOA of 0.8 cm^2^ and preserved LV-EF. A preprocedural-CCTA disclosed a severely calcified BAV Sievers type 1-a, with calcified raphe and an Agatston calcium score of 6132 and calcium volume of 4757 mm^3^ from a non-contrast CT-scan (Figure 3 and Supplementary Video 3). The measurements revealed a large annulus with a perimeter-derived diameter of 32.7 mm, a perimeter of 102.6 mm, a perimeter-derived area of 812 mm^2^, an ICD of 36 mm, wide sinuses (41 × 43 × 44 mm), and an ascending aorta of 42 mm. The left and right coronary heights were 12 and 19 mm, respectively. The decision was made to implant a 32 mm Myval-XL. A baseline ECG showed a first-degree AV block (PR interval of 222 ms) and a QRS of 104 ms. The patient was operated on through the right CFA. A Sentinel Cerebral Protection System (Boston Scientific, Marlborough, MA, USA) was inserted successfully from the right radial artery. A transvenous pacing lead through the left femoral vein was used for temporary pacing. Balloon pre-dilatation was performed using a True Dilation^®^ balloon-26 mm (Bard Peripheral Vascular, Temple, AZ) (Supplementary Video 4). The device deployment was performed using the standard delivery system (Supplementary Video 5). The final aortography revealed Sellers’ grade of 0 (Supplementary Video 6). The patient was monitored in the hospital for 7 days for any new conduction disturbance requiring permanent pacemaker implantation. Fortunately, no changes were noticed in the pre-discharge ECG. Afterward, the patient was discharged in good condition with Dabigatran 150 mg twice daily as an antithrombotic. The patient’s 30-day follow-up confirmed good clinical and hemodynamic outcomes (Table 1 and Supplementary Videos 7–9).

## Discussion

With the extension of TAVR indications into lower surgical risk and younger populations, more BAV is expected to be treated. BAV disease is associated with multiple anatomical constraints such as extensive calcifications and large anatomy (4, 5), so surgical intervention is still favored for those populations (1, 6). This case report series describes the successful implantation of a 32 mm Myval-XL and the 30-day outcome for three European patients with severe BAV stenosis and large annulus dimensions exceeding the limits of all available commercial balloon- or self-expandable transcatheter heart valves (THVs). The Myval THV received a CE mark on June 2019 and introduced five extra sizes, three intermediate (21.5, 24.5, and 27.5 mm) and two extra-large (XL) sizes (30.5 and 32 mm). The included patients are relatively young with low surgical risk, so they may become exposed to future device re-intervention (Valve-in-Valve procedure) and may benefit from a rather large second device and a lower risk of PPM. The CCTA analysis has revealed high-risk features, such as significant calcifications within the raphe and the annulus, with the risk of annular rupture, especially with BEV. Hence, the SEV is frequently selected with such features and BAV. For the three above cases, the ICD diameter was larger than the annular diameter, which revealed a flared configuration of the BAVs device landing zone.

Because the maximum limit of the native annulus area and perimeter-derived diameter for other BEVs are 683 mm^2^ and 29.5 mm, and for SEV, the maximum annulus perimeter is 94.2 mm, and the maximum perimeter-derived diameter is 30 mm, a 32 mm Myval-XL was the only THV fitting with this anatomy without the need for significant oversizing, which has been performed previously with such large anatomy (7, 8). From a technical perspective, significant oversizing might carry the risk of substantial deformation of the leaflet and stent geometry and, subsequently, lead to early structural failure, which may be less with a device designed specifically for such anatomy.

The recently introduced Navitor Titan THV (Abbott, USA) can fit larger anatomy better than other valves (9), with the limits of a 707 mm^2^ annulus area, 30 mm annulus diameter, and a perimeter of 95 mm, which is still below the range of measurements described in this report. A TAVR within large anatomy carries the risk of device instability, embolization, or the presence of significant residual PVL, especially with the significant calcifications and asymmetric valve opening of BAV, which are not encountered in the three cases in this report, emphasizing the “stable behavior” during the implantation, in addition to the acceptable post-procedural ECG changes. With the use of the 14-Fr-Python sheath, which accommodates the device through the ≥ 6.5 mm CFA diameter, only two Perclose-ProGlide (Abbott, USA) were used to successfully close the CFA without residual bleeding in the three patients. The mean procedural time and contrast amounts were 36 min and 70 ml, respectively. These findings are consistent with the initial report on Myval’s safety and efficacy in BAV (10).

## Conclusion

The implantation of the 32 mm Myval-XL device in a large annulus and BAV appears safe with promising and acceptable hemodynamic and clinical outcomes. This might be considered a step toward further expansion of the clinical indication of the TAVR practice and highlights the concept of patient-specific device selection by offering a dedicated device specifically designed for patients with large anatomy.

## Data availability statement

The original contributions presented in this study are included in the article/Supplementary material, further inquiries can be directed to the corresponding authors.

## Ethics statement

Written informed consent was obtained from the individual(s) for the publication of any potentially identifiable images or data included in this article.

## Author contributions

AE, OS, CT, and HN: conceptualization of the report and writing and drafting of the manuscript. CT, JE-G, TT, and HN: investigations. OS, CT, and HN: supervision. PS, MA, HE, JE-G, and TT: manuscript review and editing. All authors contributed to the article and approved the submitted version.

## Abbreviations

AS: Aortic Stenosis
AV: Aortic Valve
BAV: Bicuspid Aortic Valve
CCTA: Cardiac Computed Tomography Angiography
EOA: Effective Orifice Area
PVL: Paravalvular leakage
TAVR: Transcatheter Aortic Valve Replacement
TTE: Transthoracic Echocardiography.
