# Supplementary material for: Case report: Transcatheter aortic valve replacement in a large bicuspid anatomy using the XL-Myval 32 mm
Source: Front Cardiovasc Med. 2022 Nov 23;9:1045280. doi: 10.3389/fcvm.2022.1045280 (PMC9727381; doi:10.3389/fcvm.2022.1045280)
Supplement: Supplementary file 10 [file Data_Sheet_1.docx]

Supplementary Material

# Supplementary Data

Video 1: Cine-Fluoroscopy showing the deployment of Myval-XL 32mm within the annular plane for patient 1.

Video 2: Cine-Fluoroscopy showing the post-deployment of Myval-XL 32mm, final aortogram for patient 2 with sellers’ grade 1.

Video 3: CCTA (Right), Non-contrast CCT (Left) showing the calcium burden within the AV of patient 3.

Video 4: Cine-Fluoroscopy showing Balloon pre-dilatation using a True Dilation®balloon-26mm for patient 3.

Video 5: Cine-Fluoroscopy showing the post-deployment of Myval-XL 32mm final aortogram for patient 3.

Video 6: Cine-Fluoroscopy showing the post-deployment of Myval-XL 32mm final aortogram for patient 3 without residual PVL (Sellers’ grade 0).

Video 7: TTE at 30-day follow-up, parasternal long axis view showing the implanted THV with trace PVL.

Video 8: TTE at 30-day follow-up, apical five-chamber view showing the implanted THV with trace PVL.

Video 9: TTE at 30-day follow-up, apical 3 camber view showing the implanted THV with trace PVL.
